# Intraobserver and interobserver reliability of measures of cervical sagittal rotation

**DOI:** 10.1186/1471-2474-15-332

**Published:** 2014-10-04

**Authors:** Sheng-Dan Jiang, Jiang-Wei Chen, Yue-Hua Yang, Xiao-Dong Chen, Lei-Sheng Jiang

**Affiliations:** Department of Orthopaedic Surgery, Xinhua Hospital, Shanghai Jiaotong University School of Medicine, 1665 Kongjiang Road, Shanghai, 200092 China

**Keywords:** Cervical sagittal rotation, Radiographic analysis

## Abstract

**Background:**

Diagnosis and treatment decisions of cervical instability are made, in part, based on the clinician’s assessment of sagittal rotation on flexion and extension radiographs. The objective of this study is to evaluate the intraobserver and interobserver reliability of three measurement techniques in assessing cervical sagittal rotation.

**Methods:**

Fifty lateral radiographs of patients with single-level cervical degenerative disc were selected and measured on two separate occasions by three spine surgeons using three different measurement techniques. Cervical sagittal rotation was measured using three different techniques.

**Results:**

Intraclass correlation coefficients were most consistent for Method 2 (ICC 0.93-0.96) followed by Method 1 (ICC 0.88-0.91) and Method 3 (ICC 0.81-0.87). Intraobserver agreement (% of repeated measures within 0.5° of the original measurement) ranged between 76% and 96% for all techniques, with Method 2 showing the best agreement (92%-96%). Paired comparisons between observers varied considerably with interobserver reliability correlation coefficients ranging from 0.54 to 0.89. Method 2 showed the highest interobserver reliability coefficient (0.82, range 0.73-0.88). Method 2 was also more reliable for the classification of “instability”. Intraobserver percent agreements ranged from 94 to 98% for Method 2 versus 84% to 90% for Method 1 and 78% to 86% for Method 3, while interobserver percent agreements ranged from 90% to 98% for Method 2 versus 86% to 94% for Method 1 and 74% to 84% for Method 3.

**Conclusions:**

Method 2 (measuring the angle from the inferior endplate of the vertebra above the degenerative disc and the inferior endplate of the vertebra below the degenerative disc) showed the best intraobserver and interobserver reliability overall in assessing cervical sagittal rotation.

**Electronic supplementary material:**

The online version of this article (doi:10.1186/1471-2474-15-332) contains supplementary material, which is available to authorized users.

## Background

The accuracy of a method can be defined as how close a measured value is to a true value. A reliable measurement should be both accurate and precise, with precision defined by agreement between different observers and agreement for an observer who repeats the measurement several times.

Clinical instability of the cervical spine should be diagnosed accurately for clinical decision making. Flexion-extension X-rays are commonly used clinically to assess stability of the cervical spine for several medical conditions, such as trauma, post-trauma, and degeneration etc. [1–10]. Diagnosis and treatment decisions are made, in part, based on the clinician’s assessment of these X-rays. Sagittal translation (>3.5 mm), or segmental angulation (>11°) is typically used to infer instability [11], and radiographic measurements often play a pivot role in orthopaedic decision making. The steps used for the analysis of sagittal translation are well described [12]. Contrary to that, there are several techniques for the assessment of cervical sagittal rotation, and this can even be deemed to be a completely unreliable tool.

The intraobserver and interobserver variability of methods evaluating cervical sagittal rotation has not been studied. A reliability analysis is an essential step in the development of any classification system or treatment algorithm [13]. This assessment gives critical information that often leads to the modification of a proposed classification system or treatment algorithm and thus its improvement. Digital measurement has been internally precise compared with manual measurement. In this study, three spine surgeons applied three different digital measurement techniques to 50 cases with single-level degenerative disc disease to determine intraobserver and interobserver variability of cervical sagittal rotation.

## Methods

### Patients

Lateral plain radiographs of 50 cases in flexion and extension were retrieved from the institutional digital imaging system and stored on compact discs (CDs) in high quality digital tagged image file format (TIFF) for mobility measurement in cervical degenerative segment. Inclusion criterion for subjects was single-level cervical degenerative disc disease confirmed by MRI. This study was approved by the Human Research Committee of the university, and all subsequent research adhered to the 'Guidelines for Human Research' of the university. Written informed consent was obtained from the patient for the publication of this report and any accompanying images.

### Measurement procedure

Three spine surgeons who each had more than 10 years experience in spine surgery performed the measurements on computers using necessary software. Before performing the experimental measurements, each spine surgeon was trained on the use of the software and the measurement technique and demonstrated the ability to independently perform the measurements on one pair of flexion-extension radiographs. Each spine surgeon was assigned a set of radiographs and allowed to complete the measurements at his own pace over the course of 4 weeks.

**Figure 1:**
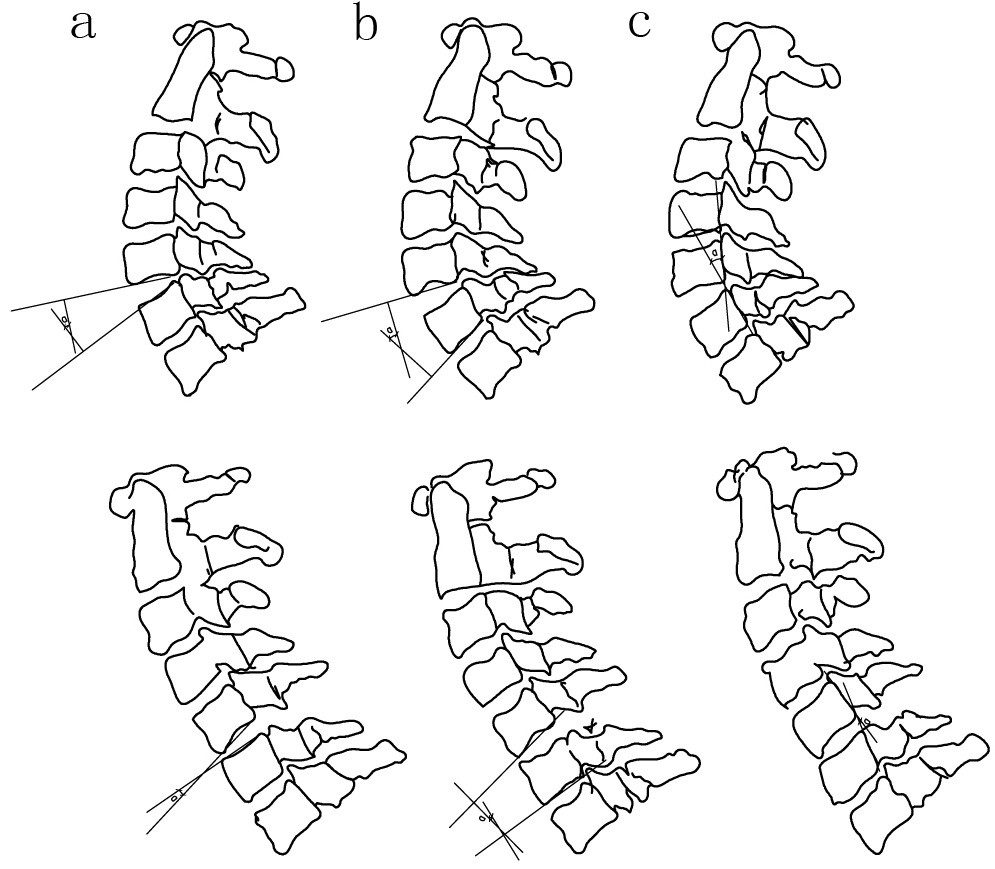
**The illustration of the lines and angles in Method 1, Method 2 and Method 3. a** Measurement of the angle from the inferior endplate of the vertebra above the degenerative disc to the superior endplate of the vertebra below on the flexion and extension films. **b** Measurement of the angle from the inferior endplate of the vertebra above the degenerative disc to that below the degenerative disc on the flexion and extension films. **c** Measurement of the angle from the posterior edge of vertebra above the degenerative disc to that below the degenerative disc on the flexion and extension films.

Each pair of digital radiographs was opened using the software. For each image, three spine surgeons measured the angle on the flexion and extension films according to three methods: Method 1; Method 2; and Method 3. Cervical sagittal rotation was defined as the change in the angulation from extension to flexion. After three weeks, three spine surgeons rated the same set of images again. The image and the order were blinded and randomized on the two occasions. Cevical sagittal rotation (>20°) was considered unstable [14]. Figure 1 illustrates the lines and angles constructed by the computer in Method 1, Method 2 and Method 3 [15–17].

### Statistical analysis

Statistical analysis was performed using SPSS 15.0 software (SPSS Inc, Chicago, IL). Three analyses were conducted in assessing the reliability of this radiographic parameter of sagittal rotation in cervical spine. Intra-class correlation coefficient (ICC) was calculated for both inter-rater and intra-rater reliability [18]. The intraobserver reliability assessed the reproducibility of each observer for each measurement technique. In this study, each observer measured the same radiograph twice for each technique. The interobserver reliabilities were obtained to assess the overall agreement among the three observers for all methods and for each method as well. For analyzing the interobserver reliability, the first measurement of each observer was entered in the ANOVA. The Pearson correlation was evaluated between the average angles estimated with different measurement techniques by all three observers in two sessions. A *P* value of less than 0.05 was considered significant. All reliability estimates were presented with a 95% confidence interval (CI).

## Results

Fifty lateral radiographs of cervical spine in flexion and extension were measured by three independent observers on two separate occasions using three different measurement techniques. MRI revealed that disc degeneration occurred at C3/4 in 11 patients, 19 cases at C4/5, 8 cases at C5/6, and 12 cases at C6/7.

The mean sagittal rotation was 9.6° (SD, 1.6°) with Method 1, 9.8° (SD, 1.5°) with Method 2, and 10.3° (SD, 1.7°) with Method 3.

### Cervical sagittal rotation measurement reliability

Reproducibility for each observer was quite high when comparing each of the three techniques (Table 1). The intraclass coefficient varied from 0.87 to 0.95 for Observer 1, 0.81 to 0.93 for Observer 2, and 0.83 to 0.96 for Observer 3. The intraclass coefficients were most consistent for Method 2 (ICC 0.93-0.96), measuring the angle from the inferior endplate of the vertebra above the degenerative disc to that below the degenerative disc. This was followed by Method 1 (ICC 0.88-0.91), measuring the angle from the inferior endplate of the vertebra above the degenerative disc to the superior endplate of the vertebra below. Method 3 (measuring the angle from the posterior edge of vertebra above the degenerative disc to that below the degenerative disc) produced the lowest intraclass coefficients of the three methods.

**Table 1:** **Comparison of the three measurements (quantitative motion analysis) using Shrout-Fleiss intraclass correlation coefficients (3,1) for intraobserver reliabilities**

Observer	Method 1	Method 2	Method 3
1	0.88 (0.79-0.95)	0.95 (0.88-0.98)	0.87 (0.79-0.94)
2	0.90 (0.84-0.95)	0.93 (0.87-0.97)	0.81 (0.70-0.89)
3	0.91 (0.85-0.96)	0.96 (0.92-0.99)	0.83 (0.74-0.91)

Intraobserver agreement (percent of repeated measures within 0.5 degree of the original measurement) ranged from 76%-96% for each technique for all three observers. The confidence interval was set at 95% (Table 2). Once again, the most consistent results overall were obtained with Method 2 (94%, 92%, and 96%). Method 3 showed the least agreement.

**Table 2:** **Probability that the same observer would measure the same radiograph within 0.5° of the initial measurement (%)**

Observer	Method 1	Method 2	Method 3
1	86 (78–95)	94 (86–98)	84 (72–92)
2	90 (81–96)	92 (83–97)	76 (67–88)
3	82 (71–91)	96 (88–99)	78 (70–90)

Using intraclass correlation coefficients for each measurement technique, paired comparisons between observers varied considerably (Table 3). Method 2 had the best interobserver reliability (ICC 0.82, CI: 0.74-0.89) and was the only method acceptable by statistical standards (0.80) as all other techniques fell well below this standard. Method 1 and Method 3 were consistently poor.

**Table 3:** **Comparison of the three measurements (quantitative motion analysis) using Shrout-Fleiss intraclass correlation coefficients (3,1) for interobserver reliabilities**

Observer	Method 1	Method 2	Method 3
All	0.71 (0.58-0.83)	0.82 (0.71-0.89)	0.62 (0.51-0.74)
1 & 2	0.75 (0.64-0.87)	0.89 (0.81-0.95)	0.54 (0.41-0.66)
1 & 3	0.72 (0.62-0.84)	0.74 (0.62-0.85)	0.67 (0.52-0.79)
2 & 3	0.61 (0.53-0.73)	0.85 (0.75-0.92)	0.65 (0.54-0.73)

There was statistically significant correlation between Method 1 and Method 2 (r = 0.982, P < 0.05), Method 1 and Method 3 (r = 0.953, P < 0.05), and Method 2 and Method 3 (r = 0.945, P < 0.05).

### Reliability of instability classification

Intraobserver reliability for the classification of instability was substantially better for Method 2 compared with the other two measurement techniques. The percentage agreement between the two ratings of instability was 98%, 94%, and 96% for Method 2, 88%, 90%, and 84% for Method 1, 86%, 82%, and 78% for Method 3 (Table 4).

**Table 4:** **Comparison of intraobserver percent agreement for classification of instability (%)**

Observer	Method 1	Method 2	Method 3
1	88 (78–95)	98 (91–100)	86 (77–94)
2	90 (82–97)	94 (87–98)	82 (72–89)
3	84 (76–93)	96 (89–99)	78 (71–86)

Method 2 also demonstrated substantially higher interobserver reliability for the classification of instability. Interobserver percent agreements ranged from 90% to 98% for Method 2 versus 86% to 94% for Method 1 and 74% to 84% for Method 3. The overall percentage agreement for the three sets of rater pairs 96% for Method 2, 90% for Method 1, and 82% for Method 3 (Table 5).

**Table 5:** **Comparison of interobserver percent agreement for classification of instability (%)**

Observer	Method 1	Method 2	Method 3
All	90 (81–96)	96 (85–99)	82 (70–91)
1 & 2	86 (76–93)	90 (82–95)	74 (62–85)
1 & 3	94 (83–98)	98 (92–100)	84 (74–92)
2 & 3	88 (79–95)	92 (87–96)	80 (69–91)

## Discussion

It was demonstrated that digital measurement was precise and Method 2 is the most reliable and least variable measurement technique. The intraobserver and interobserver reliability were markedly higher for Method 2 than the other two measurements. As expected, intraobserver reliability tended to be higher than interobserver reliability.

One of the more popular measurement techniques is the Method 1. This method measures from the inferior endplate of the vertebra above the degenerative disc to the superior endplate of the vertebra below. Our study found that this method is variable. This appears to be secondary to including a smaller area over which to measure, which maximizes differences between measurements. Method 2 appeared to have the best interobserver reliability. Maybe it is easy to establish the inferior endplate of the vertebra below the degenerative disc. Taylor et al. [19] reported that interobserver agreement (kappa = 0.17) was poor with methods routinely used in clinical practice, and computer-assisted analysis improved agreement (kappa = 0.77). To date, there is no universal agreement on how to measure cervical segmental angulation. A reliable, reproducible measurement technique is imperative to provide meaningful interstudy evaluation and comparison. This study indicates that measuring from the inferior endplate of vertebra above the degenerative disc to the inferior endplate of vertebra the below the degenerative disc is most consistent in terms of intraobserver and interobserver reliability. Recognizing the inherent limitations to any radiographic measurement, cervical stability may reliably be evaluated.

Many authors have suggested the need for instrumented fusion if instability is present, indicating that clinical decisions could be influenced by measurements of sagittal rotation. As such, we compared intra- and interobserver agreement on the classification of sagittal instability, using the criteria of 10° of rotation. Method 2 agreed with their own ratings of instability 90% to 98% of the time compared with 76% to 80% agreement for Method 3. This suggests that on two separate occasions a surgeon could arrive at different treatment decisions based on the same flexion-extension radiographs up to 22% of the time because of the imprecision of Method 2. Use of Method 2 would likely reduce this rate of disagreement to less than 10%. The pattern of interobserver agreement on instability was similar, with the percent agreement ranging from 90% to 98% for Mehod 2, compared with 70% to 80% for Method 3. This indicates that two different surgeons evaluating the same radiographs could arrive at different treatment decisions up to 30% of the time using Method 3 compared with 10% of the time using Method 2.

A reliable, reproducible methodology for evaluating stability in cervical segment is important, because this determines the modality of management. If instability is found, surgical management will be preferred. Otherwise, conservative treatment should be taken into consideration. Certain radiographic measurements, such as sagittal translation and segmental angulation in flexion and extension, are used to evaluate the stability in cervical spine. Measurement parameters should be critically examined for both validity and reliability before they can be embraced into clinical practice. To ensure applicability for all practitioners caring for patients with cervical instability, one of the first key exercises is to demonstrate its reliability. This can be a complex exercise, often appearing cumbersome and lengthy. Yet statistical analysis of this type is integral to the adoption of any treatment algorithm. This research study was such a statistical exercise in determining reliability of these parameters in the assessment of sagittal rotation in cervical spine.

There is one limitation in this study. Although C5/6 is the commonest level involved in cervical degeneration, there are more cases with C4/5 degeneration and less with C5/6 degeneration in our study.

## Conclusions

It was demonstrated that the intraobserver reliability was more consistent than interobserver reliability, regardless of the method used. Method 2 had better overall, intraobserver and interobserver reliability in assessing cervical sagittal rotation.

## Authors’ original submitted files for images

Below are the links to the authors’ original submitted files for images. Authors’ original file for figure 1
