# Supplementary figures and images for: Intraobserver and interobserver reliability of measures of cervical sagittal rotation
Source: BMC Musculoskelet Disord. 2014 Oct 4;15:332. doi: 10.1186/1471-2474-15-332 (PMC4198679; doi:10.1186/1471-2474-15-332)

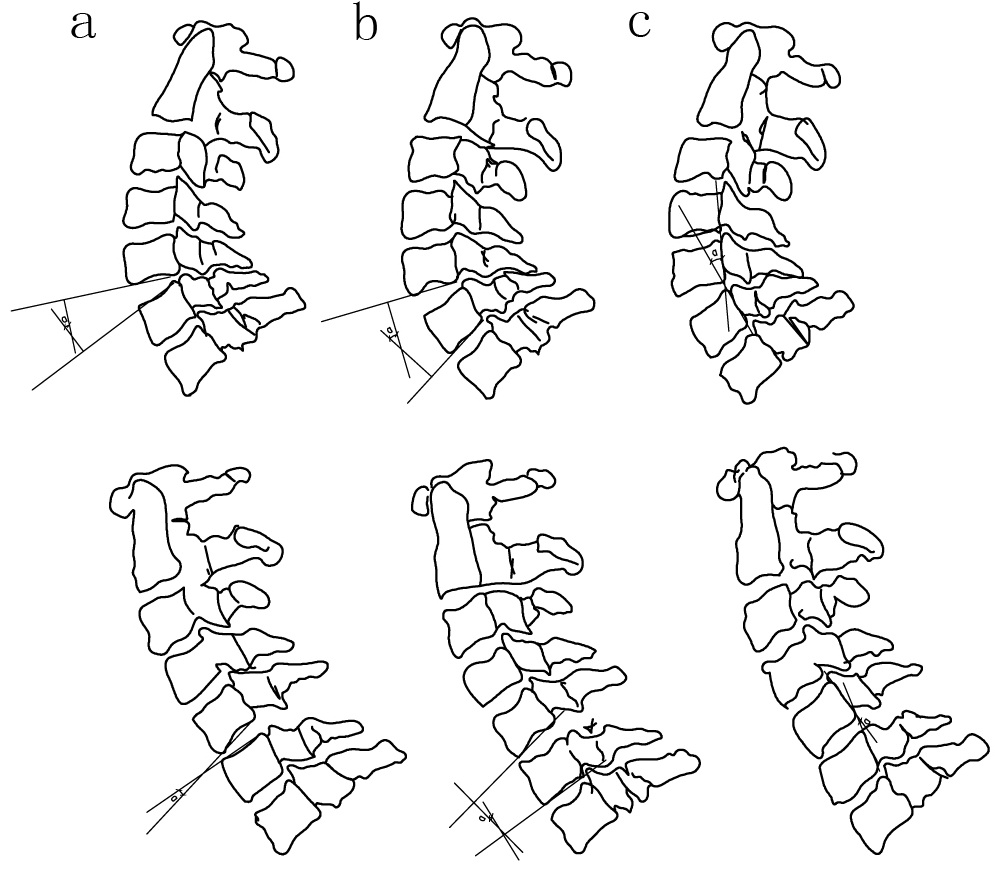

Supplement: Supplementary file 1 — Authors’ original file for figure 1 [file 12891_2013_2274_MOESM1_ESM.jpg]
